# Overexpression of MLPH in Rectal Cancer Patients Correlates with a Poorer Response to Preoperative Chemoradiotherapy and Reduced Patient Survival

**DOI:** 10.3390/diagnostics11112132

**Published:** 2021-11-17

**Authors:** Wan-Shan Li, Chih-I Chen, Hsin-Pao Chen, Kuang-Wen Liu, Chia-Jen Tsai, Ching-Chieh Yang

**Affiliations:** 1 Institute of Biomedical Sciences, National Sun Yat-sen University, Kaohsiung 804, Taiwan; a80818@mail.chimei.org.tw; 2 Department of Pathology, Chi Mei Medical Center, Tainan 710, Taiwan; 3 Department of Medical Technology, Chung Hwa University of Medical Technology, Tainan 717, Taiwan; 4 Division of Colon and Rectal Surgery, Department of Surgery, E-DA Hospital, Kaohsiung 824, Taiwan; ed106687@edah.org.tw (C.-I.C.); ed102430@edah.org.tw (H.-P.C.); ed100739@edah.org.tw (K.-W.L.); 5 Division of General Medicine Surgery, Department of Surgery, E-DA Hospital, Kaohsiung 824, Taiwan; 6 Department of Medicine, School of Medicine, I-Shou University, Kaohsiung 824, Taiwan; 7 Department of Information Engineering, I-Shou University, Kaohsiung 840, Taiwan; 8 The School of Chinese Medicine for Post Baccalaureate, I-Shou University, Kaohsiung 824, Taiwan; 9 Department of Radiation Oncology, Chi Mei Medical Center, Tainan 710, Taiwan; b101100015@tmu.edu.tw; 10 Department of Pharmacy, Chia-Nan University of Pharmacy and Science, Tainan 717, Taiwan

**Keywords:** melanophilin, rectal cancer, chemoradiotherapy, response, survival

## Abstract

Data mining of a public transcriptomic rectal cancer dataset (GSE35452) from the Gene Expression Omnibus, National Center for Biotechnology Information identified the melanophilin (*MLPH*) gene as the most significant intracellular protein transport-related gene (GO:0006886) associated with a poor response to preoperative chemoradiation. An MLPH immunostain was performed on biopsy specimens from 172 rectal cancer patients receiving preoperative chemoradiation; samples were divided into high- and low-expression groups by H-scores. Subsequently, the correlations between MLPH expression and clinicopathologic features, tumor regression grade, disease-specific survival (DSS), local recurrence-free survival (LRFS), and metastasis-free survival (MeFS) were analyzed. MLPH expression was significantly associated with CEA level (*p* = 0.001), pre-treatment tumor status (*p* = 0.022), post-treatment tumor status (*p* < 0.001), post-treatment nodal status (*p* < 0.001), vascular invasion (*p* = 0.028), and tumor regression grade (*p* < 0.001). After uni- and multi-variable analysis of five-year survival, MLPH expression was still associated with lower DSS (hazard ratio (HR), 10.110; 95% confidence interval (CI), 2.178–46.920; *p* = 0.003) and MeFS (HR, 5.621; 95% CI, 1.762–17.931; *p* = 0.004). In conclusion, identifying MLPH expression could help to predict the response to chemoradiation and survival, and aid in personal therapeutic modification.

## 1. Introduction

Colorectal cancer remains among the most frequently diagnosed cancers in the world, with significant cancer-associated mortality [1]. On the basis of current evidence, the standard treatment for patients with advanced rectal cancer involves surgery with preoperative radiation with or without chemotherapy [2]. These advanced therapeutic options have improved patient survival. However, 10–20% of patients still experience recurrence or metastasis after initial treatment [3]. Although these rectal cancer patients with tumor failure may be eligible for salvage treatment, they still face a dismal outcome. Therefore, it is important to find reliable genetic biomarkers as predictors of the response to preoperative therapy and prognosis, and to aid in personal therapeutic modification.

The intracellular transport of cellular proteins and organelles is critical for maintaining the structural and functional integrity of the cell, enabling the various subcellular compartments to carry out their unique metabolic roles [4,5]. The current literature reports that changes in the expression of these proteins have been associated with cancer invasion, metastasis, and survival [6,7]. Therefore, to identify the potential biomarkers related to intracellular protein transport in rectal cancer, a public transcriptomic dataset of rectal cancer (GSE35452) from the Gene Expression Omnibus, National Center for Biotechnology Information (GEO, NCBI, Bethesda, MD, USA) was used and melanophilin (*MLPH*) was recognized as the most significantly up-regulated gene (GO:0006886).

Therefore, our current study aimed to investigate the association between MLPH expression and clinical outcomes, including tumor response to preoperative chemoradiation and survival, in our 172 rectal cancer patients treated with preoperative chemoradiation.

## 2. Materials and Methods

### 2.1. Data Mining of the Published Transcriptomic Rectal Cancer Dataset

In the NCBI GEO public transcriptomic database (GSE35452), information from 46 rectal cancer patients who had undergone preoperative chemoradiation was used for data mining. To quantify the expression levels, the raw. cel files on the Affymetrix Human Genome U133 Plus 2.0 microarray platform (Affymetrix, Inc., Santa Clana, CA, USA) were analyzed using the Nexus Expression 3 (BioDiscovery, Hawthorne, CA, USA) software, employing a comparative analysis without filtering or preselection. According to the response to preoperative chemoradiation, the samples were separated into “responders” and “nonresponders”, with special attention given to the genes involved in intracellular protein transport (GO:0006886). Transcripts with *p* < 0.01 and an expression fold change > ±0.1 log2 ratio were selected for analysis.

### 2.2. Patients and Tumor Characteristics

In this study, patients with newly diagnosed rectal adenocarcinoma between 1998 and 2004 were identified from the cancer registry database of Chi Mei Medical Center. The clinical staging was based on abdominal computed tomography (CT) or magnetic resonance imaging (MRI), and was revised according to the 7th edition of the American Joint Committee on Cancer (AJCC) staging system. All patients received standardized long-course chemoradiation, including 5-fluorouracil-based chemotherapy either orally or intravenously every week from the commencement of radiation therapy to completion. The radiation dose ranged from 45–50 Gy in 25 fractions and the whole course usually ran over a 5-week period. Radical surgery with total mesorectal excision (abdominoperineal resection or low anterior resection) was performed 4–6 weeks after chemoradiotherapy. Approximately 3 to 4 weeks after surgery, 5-fluorouracil-based adjuvant chemotherapy was administered for at least 4 months if the pre-treatment or post-treatment tumor or nodal status was beyond T3 or N1, respectively. Patients with incomplete clinicopathological information or a history of either cancer or metastatic disease were excluded. Finally, a total of 172 rectal cancer patients who were regularly followed up on until death or the final follow-up were included, and their formalin-fixed paraffin-embedded (FFPE) tissue specimens were used for analysis. The data extracted from the medical records included the date of diagnosis, age, gender, clinical/pathological characteristics (such as location, CEA level, stage, lymphovascular invasion, perineural invasion, and chemotherapy use), and cause of death, if relevant.

### 2.3. Histopathologic and Immunohistochemical Analysis

Tumor FFPE specimens were evaluated by a pathologist blinded to the patients’ clinical information. The extent of tumor regression (tumor regression grade, TRG) after preoperative chemoradiation was assessed using a five-point system, and the study patients were classified as having: a poor response with TRG 0–1 (<25% response); a moderate response (TRG 2–3); or no visible tumor in the rectal wall with TRG 4 (complete response) [8]. As our previous studies described, the method of immunohistochemistry included deparaffinized, rehydrated, heated, quenched, and washed for pretreatment rectal tumor biopsy specimens [9,10]. Subsequently, the tissue sections were incubated for 1 h with a primary antibody recognizing MLPH (ThermoFisher Scientific, Waltham, MA, USA; Clone: OTI6E3, 1:200). The H-score was calculated to interpret the MLPH protein expression and was quantified using the following equation: H-score = Σpi (I + 1), where Pi is the percentage of stained tumor cells for each intensity (0–100%), and i represents the intensity of staining (0 to 3+) [11]. The definition of high and low MLPH expression were as follows: high expression, above or equal to the median; and low expression, below the median of all scored cases.

### 2.4. Statistical Analysis

The chi-square test was used to determine the association between MLPH expression and clinicopathological characteristics. The Kaplan–Meier method was applied to estimate our endpoints: the 5-year disease-specific survival (DSS), local recurrence-free survival (LRFS), and metastasis-free survival (MeFS), with the log-rank test used for comparison. Recurrence or deaths due to cancer were defined as events. All factors were analyzed using univariate Cox regression to estimate hazard ratios (HRs) with 95% confidence intervals (CIs). Those factors with statistical significance were entered into multivariate analysis. All statistical analyses and graphics were executed using SPSS for Windows 22.0 (IBM Corporation, Armonk, NY, USA). A *p* value less than 0.05 was determined to be statistically significant.

## 3. Results

### 3.1. Up-Regulation of the MLPH Gene Is the Most Significant Intracellular Protein Transport Factor Related to Preoperative Chemoradiotherapy

**Figure 1:**
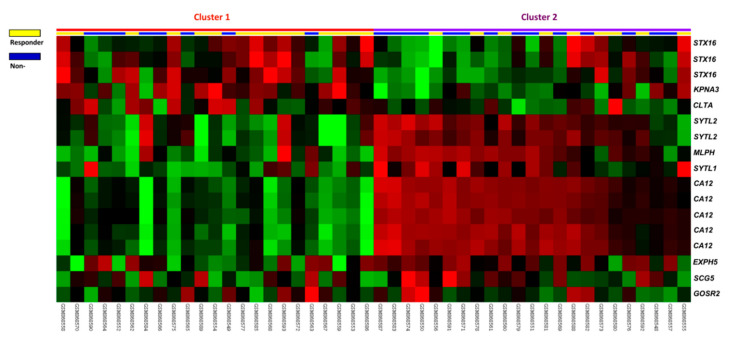
Analysis of gene expression in chemoradiation responders versus non-responders from a published transcriptomic dataset of rectal cancers (GSE35452). Tissue specimens from responders (yellow lines) and non-responders (blue lines) are indicated on top of the heatmap, and expression levels of up-regulated and down-regulated genes are expressed as a spectrum of brightness of red and green. Melanophilin (MLPH) was identified as the most significantly upregulated gene related to intracellular protein transport (GO:0006886).

As described above, a public transcriptomic dataset (GSE35452) that included 46 rectal cancer patients was analyzed to find potential biomarkers, focusing on intracellular protein transport (GO: 0006886). As shown in Table 1 and Figure 1, MLPH was the most significantly up-regulated gene associated with a poor response to preoperative chemoradiotherapy (log 2 ratio, 1.2845; *p* < 0.0001). This finding prompted an investigation of the relationship between MLPH expression and clinicopathologic behavior in rectal cancers after chemoradiation. 

### 3.2. Clinicopathological Characteristics of Study Patients 

Table 2 details the characteristics of the 172 patients with rectal adenocarcinoma treated with preoperative chemoradiotherapy followed by radical surgery. Most of these patients were men (108, 62.8%) and the median age was 63 years (range, 22–88 years). AJCC tumor staging identified 41.9% of patients at stage I, 29.9% at stage II, and 28.1% at stage III disease. Patients with clinical or pathological stage II or III disease received adjuvant chemotherapy. In terms of other pathological features, 15 (8.7%) patients presented with vascular invasion and 5 (2.9%) with perineurial invasion. After chemoradiation, 17 (10%) patients had complete tumor regression (TRG 4), 118 (68.6%) patients had a moderate response (TRG 2–3), and 37 (21.5%) patients had a poor response (TRG 0–1). In the Supplementary Figure S1, TRG was statistically significantly correlated with 5-year DSS, LRFS, and MeFS. For patients with a complete response (TRG 4), the 5-year DSS was higher than TRG 2+3 and TRG 0+1 (92% versus 76% versus 42%, *p* < 0.05). They were also statistically significant in 5-year LRFS and MeFS (*p* < 0.05).

### 3.3. Immunohistochemical Analysis

**Figure 2:**
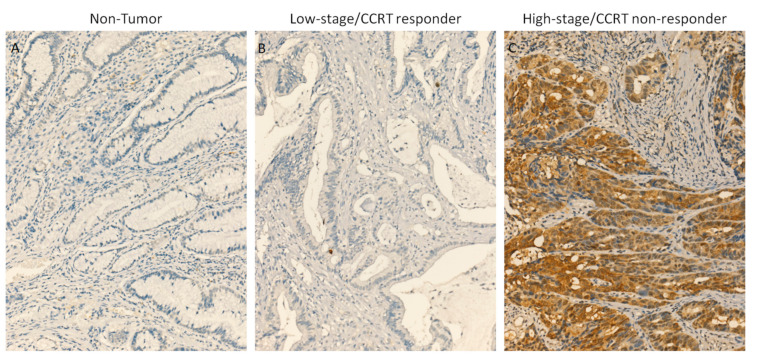
Immunohistochemical staining of melanophilin (MLPH) expression in: (**A**) normal mucosa: no MLPH expression; (**B**) rectal carcinoma responsive to chemoradiation: low MLPH expression; and (**C**) rectal carcinoma unresponsive to chemoradiation: high MLPH expression.

After dividing our rectal cancer patients into groups with high and low MLPH expression, immunohistochemical staining was performed to assess the relationship of this factor with clinicopathologic characteristics (Figure 2). Cytoplasmic expression of MLPH was successfully scored in all examined cases with a wide range of H-scores, varying from 100 to 290. As shown in Table 2, high MLPH expression was significantly associated with CEA ≥ 5 (*p* = 0.001), pre-treatment tumor status (T3–4; *p* = 0.022), post-treatment tumor status (T3–4; *p* < 0.001), post-treatment nodal status (N1–2; *p* < 0.001), vascular invasion (*p* = 0.028), and poor response to preoperative chemoradiotherapy (*p* < 0.001). In the group with high MLPH expression, we also observed a tumor regression grade of 0–1 in 30 (17.4%) patients, grade 2–3 in 51 (29.7%) patients, and grade 4 in 5 (2.9%) patients, indicating that MLPH expression plays an important role in the aggressive behavior of rectal tumors and their sensitivity to chemoradiation.

### 3.4. Prognostic Significance of MLPH Expression

**Figure 3:**
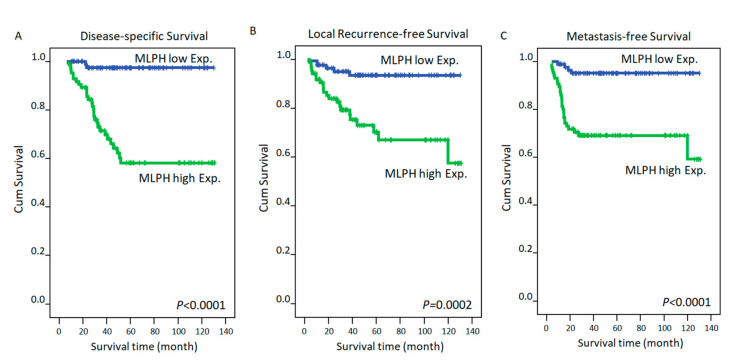
Kaplan-Meier survival between high and low melanophilin (MLPH) in 172 rectal cancer patients: (**A**) disease-specific survival; (**B**) recurrence-free survival; and (**C**) metastasis-free survival.

In Table 3, the univariate analysis indicated that clinicopathological variables, such as post-treatment tumor status and TRG, were significantly associated with a worse DSS, LRFS, and MeFS rate (all *p* ≤ 0.05). Location from anal verge was significantly associated with DSS (0.0473), and pre-treatment nodal status was significantly only related to LRFS (*p* = 0.007). CEA level (≥5 ng/mL) and presence of vascular invasion were negatively associated with DSS and LRFS to statistical significance (*p* ≤ 05 for all). Most importantly, rectal cancer patients with MLPH overexpression had significantly lower DSS (*p* < 0.0001), LRFS (*p* = 0.0002), and MeFS (*p* < 0.0001), as shown in Table 3 and Figure 3. In multivariate analyses (Table 4), MLPH expression was still associated with lower DSS (hazard ratio (HR), 10.110; 95% confidence interval (CI), 2.178–46.920; *p* = 0.003) and MeFS (HR, 5.621; 95% CI, 1.762–17.931; *p* = 0.004).

## 4. Discussion

Previous studies have revealed that MLPH is associated with tumor development, progression, and metastasis in many cancer types [12,13,14,15]. However, its role in rectal cancer, especially in terms of the response to chemoradiation, remains unknown. By considering the predisposing clinicopathological factors, our study observed that MLPH overexpression is associated with advanced tumor status, poor response to chemoradiation, and worse survival. These findings indicate that MLPH may be a potential biomarker to predict survival and a promising therapeutic target in the treatment of rectal cancer.

Tumor response after preoperative chemoradiation in rectal cancer varies considerably. Previous studies have demonstrated that down-staging or even the complete disappearance of the malignant cells in the rectal wall and perirectal nodes is associated with better survival [8,16]. Therefore, an accurate evaluation of the tumor response to preoperative chemoradiation is essential for predicting clinical outcomes and developing further treatment plans. Many techniques are used to evaluate the response after preoperative treatment: endorectal ultrasounds, CT scans, MRIs, or positron emission tomography scans [17,18,19,20]. However, these imaging systems cannot provide accurate information on the stage of the tumor (T or N categories) when they are compared with preoperative images. An alternative method of evaluation includes assessing levels of carcinoembriogenic antigen or other molecular markers [21,22,23]. No conclusion has yet been reached as to which factor or marker (e.g., Ki-67, p21, p53, or Cox-2) is representative of cancer progress, due to the small sample size of the majority of these studies [24,25,26]. Thus, we sought to provide a better marker for disease progression.

Data mining of a public transcriptomic rectal cancer dataset (GSE35452) from the NCBI GEO identified the *MLPH* gene as the most significantly intracellular protein transport-related gene (GO:0006886) associated with poor response to preoperative chemoradiotherapy. The *MLPH* gene is known to encode a member of the exophilin subfamily of Rab effector proteins [27]. The protein forms a ternary complex with the small Ras-related GTPase Rab27A in its GTP-bound form and the motor protein myosin Va [28]. MLPH is an important component of the melanosome transport mechanism, which is involved in the visible pigmentation of hair and skin. Diseases associated with MLPH include Griscelli syndrome type 3, which is characterized by a silver-gray hair color and abnormal pigment distribution in the hair shaft [29].

In recent decades, MLPH had been found to be associated with cancer. Many studies have demonstrated that MLPH contributes to tumor development, behavior, and metastasis in skin, breast, and prostate cancer [14,15,30]. Using The Human Protein Atlas database, Guo et al. found that MLPH expression was significantly up-regulated in malignant melanomas compared with benign nevi and normal skin [30]. Moreover, moderate to high melanophilin expression has been commonly observed in patients with advanced-stage melanoma. In breast cancer, Thakkar et al. found *MLPH* gene expression in aggressive tumors (basal-like subtypes) [14]. Another study noted downregulation of the *MLPH* gene in lymph node-positive breast cancer patients [12]. Zhang et al. also observed that MLPH expression is related to cell migration, proliferation, and invasion in prostate cancer [15]. All of these studies support our findings that higher MLPH expression is significantly associated with advanced tumor behavior, including pre-treatment tumor status, post-treatment tumor status, post-treatment nodal status, and vascular invasion. Therefore, altered MLPH biology is likely to play an important role in colorectal tumor formation and progression.

Although the association between MLPH expression and colorectal cancer outcomes remains unknown, our study is the first to demonstrate that a high expression of MLPH is associated with a poor response to preoperative chemoradiation and confers a negative impact on survival (DSS, LRFS, and MeFS). Similarly, Guo et al. observed that MLPH expression is altered in melanoma patients, and high levels of MLPH expression are associated with poor survival (*p* = 0.02), based on data from The Cancer Genomics Atlas database [30]. Zhang et al. similarly observed a decreasing trend in the overall survival of the patients with prostate cancer who had a high expression of MLPH [15]. Collectively, these data indicate that high MLPH expression is not only associated with a more aggressive cancer phenotype, but also acts as a predictor of poor prognosis.

More importantly, we found that high MLPH expression was also significantly associated with lower TRG scores, indicating a poor response to preoperative chemoradiation. The possible mechanisms for this association are as follows. First, transforming growth factor beta (TGF-β) is a key downstream effector of MLPH [31]. TGF-β is known to modulate late post-radiation changes, such as DNA repair, cell cycle progression, inflammation at an early stage, and the later development of fibrosis [32]. Aberrant MLPH expression is associated with an inhibition of the regulation of TGF-β and the decreased production of profibrotic growth factors, leading to a poor response to radiation [33]. Second, MLPH also acts as an effector protein for Rab27, which has long been implicated in the progression of various cancers. Recent studies have reported that Rab27 plays a significant role in the resistance to chemotherapy or radiation in many types of cancer [34,35]. Thus, MLPH expression is involved in the resistance to radiotherapy or chemotherapy, leading to lower odds of survival.

There are some limitations to this study that need to be addressed. This study was analyzed using a public transcriptomic dataset and tumor tissues from our cohort. We did not verify the MLPH mRNA or protein levels through PCR or Western blotting, which may further support our findings regarding chemoradiation therapy. Second, we included some early-stage rectal cancer patients who were receiving preoperative chemoradiation for organ preservation. Therefore, we were able to comprehensively observe the association between MLPH expression and early-/advanced-stage groups and their tumor regression grade. Finally, our study lacks information on factors such as mesorectal fascia involvement, KRAS, BRAF, and MMR status of the tumors. Future studies that include these factors are necessary to confirm our results.

## 5. Conclusions

This study is the first to reveal that MLPH expression can be a prognostic marker for rectal cancer outcomes. Our results indicate that high levels of MLPH expression are associated with significantly aggressive tumor behavior, poor response to chemoradiation, and shorter survival. Therefore, further study to better understand the molecular mechanisms of MLPH could provide effective insights for rectal cancer treatment. Studies should also be undertaken to determine whether MLPH expression could serve as an effective marker of prognosis in order to better guide clinical treatment plans.

## Figures and Tables

**Table 1:** Summary of differentially expressed genes associated with intracellular protein transport (GO: 0006886) in relation to response to chemoradiation in rectal carcinoma.

Probe	Comparison Log Ratio	Comparison *p*-Value	Gene Symbol	Gene Name	Biological Process	Molecular Function
218211_s_at	1.2845	<0.0001	*MLPH*	melanophilin	intracellular protein transport, melanocyte differentiation, melanosome localization, pigmentation, protein targeting	Rab GTPase binding, actin binding, metal ion binding, microtubule plus-end binding, myosin V binding, myosin binding, protein binding, zinc ion binding
210735_s_at	1.0681	0.0015	*CA12*	carbonic anhydrase XII	one-carbon compound metabolic process	carbonate dehydratase activity, lyase activity, metal ion binding, zinc ion binding
203963_at	1.0002	<0.0001				
204508_s_at	0.9739	0.0015				
215867_x_at	0.7435	0.0009				
214164_x_at	0.6384	0.0007				
214734_at	0.7604	<0.0001	*EXPH5*	exophilin 5	intracellular protein transport	Rab GTPase binding, protein binding
203889_at	0.6559	0.0005	*SCG5*	secretogranin V (7B2 protein)	intracellular protein transport, neuropeptide signaling pathway, peptide hormone processing, protein folding, regulation of hormone secretion, transport	GTP binding, enzyme activator activity, enzyme inhibitor activity, protein binding, unfolded protein binding
221638_s_at	−0.567	0.0059	*STX16*	syntaxin 16	intra-Golgi vesicle-mediated transport, intracellular protein transport, protein transport, proteolysis, transport, vesicle-mediated transport	SNAP receptor activity, aminopeptidase activity, hydrolase activity, manganese ion binding, metal ion binding, peptidase activity, protein binding, protein transporter activity, zinc ion binding
1558249_s_at	−0.5013	0.0042				
221499_s_at	−0.4584	0.0096				
225496_s_at	0.5602	0.0062	*SYTL2*	synaptotagmin-like 2	intracellular protein transport, transport, vesicle-mediated transport	Rab GTPase binding, neurexin binding, phosphopantetheine binding, protein binding, transporter activity, zinc ion binding
232914_s_at	0.4675	0.0025				
227134_at	0.513	0.0067	*SYTL1*	synaptotagmin-like 1	ATP synthesis coupled proton transport, intracellular protein transport, transport, vesicle-mediated transport	ATP binding, Rab GTPase binding, hydrogen ion transporting ATP synthase activity; rotational mechanism, hydrogen ion transporting ATPase activity; rotational mechanism, neurexin binding, protein binding, transporter activity
221503_s_at	−0.4765	0.005	*KPNA3*	karyopherin alpha 3 (importin alpha 4)	NLS-bearing substrate import into nucleus, intracellular protein transport, protein complex assembly, protein import into nucleus, protein transport, transport	binding, nuclear localization sequence binding, protein binding, protein transporter activity
1560434_x_at	−0.1892	0.0062	*CLTA*	clathrin; light chain (Lca)	intracellular protein transport, protein complex assembly, vesicle-mediated transport	calcium ion binding, protein binding, protein transporter activity, structural molecule activity
243880_at	0.1621	0.0065	*GOSR2*	Golgi SNAP receptor complex member 2	ER to Golgi vesicle-mediated transport, intracellular protein transport, membrane fusion, protein transport, transport, vesicle-mediated transport	receptor activity, transporter activity

**Table 2:** Associations and comparisons between MLPH expression and clinicopathological factors in 172 rectal cancer patients receiving neoadjuvant chemoradiotherapy.

Parameter		No.	MLPH Expression		*p*-Value
			Low Exp	High Exp	
Gender	Male	108	50	58	0.269
	Female	64	36	28	
Age (years)	<70	106	48	58	0.117
	≥70	66	38	28	
Location from anal verge (cm)	<6	79	44	35	0.168
	≥6	93	42	51	
CEA level (ng/mL)	<5	114	67	47	0.001 *
	≥5	58	19	39	
Pre-Tx tumor status (Pre-T)	T1–T2	81	48	33	0.022 *
	T3–T4	91	38	53	
Pre-Tx nodal status (Pre-N)	N0	125	63	62	0.864
	N1–N2	47	23	24	
Post-Tx tumor status (Post-T)	T1–T2	86	67	19	<0.001 *
	T3–T4	86	19	67	
Post-Tx nodal status (Post-N)	N0	123	73	50	<0.001 *
	N1–N2	49	13	36	
Vascular invasion	Absent	157	83	74	0.028 *
	Present	15	3	12	
Perineurial invasion	Absent	167	85	82	0.368
	Present	5	1	4	
Tumor regression grade	Grade 0–1	37	7	30	<0.001 *
	Grade 2~3	118	67	51	
	Grade 4	17	12	5	

* statistically significant.

**Table 3:** Univariate log-rank analysis for important clinicopathological variables and MLPH expression.

Parameter		No. of Case	DSS		LRFS		MeFS	
			No. of Event	*p*-Value	No. of Event	*p*-Value	No. of Event	*p*-Value
Gender	Male	108	20	0.9026	7	0.2250	17	0.3520
	Female	64	11		20		14	
Age	<70	106	19	0.8540	18	0.6615	20	0.7427
	≥70	66	12		9		11	
Location from anal verge (cm)	<6	79	8	0.0473 *	9	0.2411	15	0.7514
	≥6	93	23		18		16	
CEA level (ng/mL)	<5	114	15	0.0216 *	13	0.0179 *	17	0.1460
	≥5	58	16		14		14	
Pre-Tx tumor status (Pre-T)	T1–T2	81	10	0.0776	10	0.2261	11	0.1745
	T3–T4	91	21		17		20	
Pre-Tx nodal status (Pre-N)	N0	125	19	0.0711	15	0.0070 *	19	0.0973
	N1–N2	47	21		12		12	
Post-Tx tumor status (Post-T)	T1–T2	86	7	0.0006 *	7	0.0040 *	8	0.0033 *
	T3–T4	86	24		20		23	
Post-Tx nodal status (Post-N)	N0	123	21	0.5998	16	0.1320	20	0.4634
	N1-N2	49	10		11		11	
Vascular invasion	Absent	157	25	0.0184 *	21	0.0028 *	27	0.4470
	Present	15	6		6		4	
Perineurial invasion	Absent	167	29	0.2559	25	0.0940	30	0.9083
	Present	5	2		2		1	
Tumor regression grade	Grade 0–1	37	13	0.0038 *	10	0.0090 *	14	0.0006 *
	Grade 2~3	118	17		17		16	
	Grade 4	17	1		0		1	
Down-stage after CCRT	Non-Sig.	150	29	0.1651	24	0.5961	30	0.0853
	Sig. (≥2)	22	2		3		1	
MLPH expression	Low Exp.	86	2	<0.0001 *	5	0.0002 *	4	<0.0001 *
	High Exp.	86	29		22		27	

DSS, disease-specific survival; LRFS, local recurrence-free survival; MeFS, metastasis-free survival; * statistically significant.

**Table 4:** Multivariate analysis.

Parameter	DSS			LRFS			MeFS		
	HR	95% CI	*p*-Value	HR	95% CI	*p*-Value	HR	95% CI	*p*-Value
Tumor regression grade	1.682	0.836–3.387	0.145	2.653	1.216–5.793	0.014 *	2.037	1.046–7.937	0.036 *
MLPH expression	10.110	2.178–46.920	0.003 *	2.372	0.764–7.367	0.135	5.621	1.762–17.931	0.004 *
Vascular invasion	2.267	0.870–5.910	0.094	4.572	1.570–13.315	0.005 *	-	-	-
Post-Tx tumor status (Post-T)	1.213	0.500–2.938	0.669	1.323	0.502–3.482	0.571	1.138	0.473–2.738	0.772
Pre-Tx nodal status (Pre-N)	-	-	-	2.456	1.069–5.647	0.034 *		-	-
Location from anal verge (cm)	1.653	0.721–3.787	0.235	-	-	-			
CEA level (ng/mL)	1.615	0.773–3.375	0.202	0.786	0.338–1.828	0.576			

DSS, disease-specific survival; LRFS, local recurrence-free survival; MeFS, metastasis-free survival; * statistically significant.

## Data Availability

A public transcriptomic dataset from the Gene Expression Omnibus database (National Center for Biotechnology Information, Bethesda, MD, USA) (GSE35452) was used. Clinicopathological datasets are available from the corresponding author upon reasonable request.

## Supplementary Materials

The following are available online at https://www.mdpi.com/article/10.3390/diagnostics11112132/s1, Figure S1: Kaplan-Meier curves for 5-year disease free survival (DFS), local recurrence free survival (LRFS) and metastases free survival (MeFS) in relation to tumor regression grading (TRG), (TRG 4 vs. TRG2+3 vs. TRG 0+1).

## Abbreviations

GEO	Gene Expression Omnibus
NCBI	National Center for Biotechnology Information
MLPH	melanophilin
CT	computed tomography
MRI	magnetic resonance imaging
AJCC	American Joint Committee on Cancer
FFPE	formalin-fixed paraffin-embedded
TRG	tumor regression grade
DSS	disease-specific survival
LRFS	local recurrent-free survival
MeFS	metastasis-free survival
HR	hazard ratio
CI	confidence interval
TGF-β	transforming growth factor beta

## References

[1] Siegel R.L., Miller K.D., Jemal A. (2019). Cancer statistics, 2019. CA Cancer J. Clin..

[2] Sauer R., Becker H., Hohenberger W., Rodel C., Wittekind C., Fietkau R., Martus P., Tschmelitsch J., Hager E., Hess C.F. (2004). Preoperative versus Postoperative Chemoradiotherapy for Rectal Cancer. N. Engl. J. Med..

[3] Van den Brink M., Stiggelbout A.M., van den Hout W.B. (2004). Clinical nature and prognosis of locally recurrent rectal cancer after total mesorectal excision with or without preoperative radiotherapy. J. Clin. Oncol..

[4] Rothman J.E. (1994). Mechanisms of intracellular protein transport. Nat. Cell Biol..

[5] Rehling P., Rospert S. (2010). Molecular chaperones and intracellular protein transport. Biochim. Biophys. Acta BBA Bioenerg..

[6] Ghosh S., Shinogle H.E., Galeva N.A., Dobrowsky R.T., Blagg B.S.J. (2016). Endoplasmic Reticulum-resident Heat Shock Protein 90 (HSP90) Isoform Glucose-regulated Protein 94 (GRP94) Regulates Cell Polarity and Cancer Cell Migration by Affecting Intracellular Transport. J. Biol. Chem..

[7] Becker H., Deitmer J. (2021). Proton Transport in Cancer Cells: The Role of Carbonic Anhydrases. Int. J. Mol. Sci..

[8] Rödel C., Martus P., Papadoupolos T., Füzesi L., Klimpfinger M., Fietkau R., Liersch T., Hohenberger W., Raab R., Sauer R. (2005). Prognostic Significance of Tumor Regression After Preoperative Chemoradiotherapy for Rectal Cancer. J. Clin. Oncol..

[9] Li C.-F., He H.-L., Wang J.-Y., Huang H.-Y., Wu T.-F., Hsing C.-H., Lee S.-W., Lee H.-H., Fang J.-L., Huang W.-T. (2014). Fibroblast growth factor receptor 2 overexpression is predictive of poor prognosis in rectal cancer patients receiving neoadjuvant chemoradiotherapy. J. Clin. Pathol..

[10] Sheu M.-J., Li C.-F., Lin C.-Y., Lee S.-W., Lin L.-C., Chen T.-J., Ma L.-J. (2014). Overexpression of ANXA1 confers independent negative prognostic impact in rectal cancers receiving concurrent chemoradiotherapy. Tumor Biol..

[11] Rezaeian A.-H., Li C.-F., Wu C.-Y., Zhang X., DeLacerda J., You M.J., Han F., Cai Z., Jeong Y.S., Jin G. (2017). A hypoxia-responsive TRAF6–ATM–H2AX signalling axis promotes HIF1α activation, tumorigenesis and metastasis. Nat. Cell Biol..

[12] Abba M., Sun H., Hawkins K.A., Drake J.A., Hu Y., Nunez M.I., Gaddis S., Shi T., Horvath S., Sahin A. (2007). Breast Cancer Molecular Signatures as Determined by SAGE: Correlation with Lymph Node Status. Mol. Cancer Res..

[13] Orgaz J.L., Benguria A., Sanchez-Martinez C., Ladhani O., Volpert O.V., Jimenez B. (2011). Changes in the gene expression profile of A375 human melanoma cells induced by overexpression of multifunctional pigment epithelium-derived factor. Melanoma Res..

[14] Thakkar A.D., Raj H., Chakrabarti D., Ravishankar, Saravanan N., Muthuvelan B., Balakrishnan A., Padigaru M. (2010). Identification of Gene Expression Signature in Estrogen Receptor Positive Breast Carcinoma. Biomark. Cancer.

[15] Zhang T., Sun Y., Zheng T., Wang R., Jia D., Zhang W. (2020). MLPH Accelerates the Epithelial–Mesenchymal Transition in Prostate Cancer. OncoTargets Ther..

[16] Bouzourene H., Bosman F.T., Seelentag W., Matter M., Coucke P. (2002). Importance of tumor regression assessment in predicting the outcome in patients with locally advanced rectal carcinoma who are treated with preoperative radiotherapy. Cancer.

[17] Williamson P.R., Hellinger M.D., Larach S.W., Ferrara A. (1996). Endorectal ultrasound of T3 and T4 rectal cancers after preoperative chemoradiation. Dis. Colon Rectum.

[18] Kwok H., Bissett I.P., Hill G.L. (2000). Preoperative staging of rectal cancer. Int. J. Colorectal Dis..

[19] Brown G., Radcliffe A.G., Newcombe R.G., Dallimore N.S., Bourne M.W., Williams G.T. (2003). Preoperative assessment of prognostic factors in rectal cancer using high-resolution magnetic resonance imaging. Br. J. Surg..

[20] Hodgman C.G., Maccarty R.L., Wolff B.G., May G.R., Berquist T.H., Sheedy P.F., Beart R.W., Spencer R.J. (1986). Preoperative staging of rectal carcinoma by computed tomography and 0.15T magnetic resonance imaging. Dis. Colon Rectum.

[21] Yang K.-L., Yang S.-H., Liang W.-Y., Kuo Y.-J., Lin J.-K., Lin T.-C., Chen W.-S., Jiang J.-K., Wang H.-S., Chang S.-C. (2013). Carcinoembryonic antigen (CEA) level, CEA ratio, and treatment outcome of rectal cancer patients receiving pre-operative chemoradiation and surgery. Radiat. Oncol..

[22] Ishihara S., Watanabe T., Kiyomatsu T., Yasuda K., Nagawa H. (2010). Prognostic significance of response to preoperative radiotherapy, lymph node metastasis, and CEA level in patients undergoing total mesorectal excision of rectal cancer. Int. J. Colorectal Dis..

[23] Park Y.J., Oh B.R., Lim S.W., Huh J.W., Joo J.K., Kim Y.J., Kim H.R. (2010). Clinical Significance of Tumor Regression Grade in Rectal Cancer with Preoperative Chemoradiotherapy. J. Korean Soc. Coloproctol..

[24] Smith F., Reynolds J., Miller N., Stephens R., Kennedy M. (2006). Pathological and molecular predictors of the response of rectal cancer to neoadjuvant radiochemotherapy. Eur. J. Surg. Oncol..

[25] Moureau-Zabotto L., Farnault B., de Chaisemartin C., Esterni B., Lelong B., Viret F., Giovannini M., Monges G., Delpero J.-R., Bories E. (2011). Predictive Factors of Tumor Response After Neoadjuvant Chemoradiation for Locally Advanced Rectal Cancer. Int. J. Radiat. Oncol..

[26] Scott N., Hale A., Deakin M., Hand P., Adab F., Hall C., Williams G., Elder J. (1998). A histopathological assessment of the response of rectal adenocarcinoma to combination chemo-radiotherapy: Relationship to apoptotic activity, p53 and bcl-2 expression. Eur. J. Surg. Oncol..

[27] Xu J., Xie M., Zou S., Liu X., Li X., Xie J., Zhang X. (2016). Interactions of allele E of the MC1R gene with FM and mutations in the MLPH gene cause the five-gray phenotype in the Anyi tile-like gray chicken. Genet. Mol. Res..

[28] Nagashima K., Torii S., Yi Z., Igarashi M., Okamoto K., Takeuchi T., Izumi T. (2002). Melanophilin directly links Rab27a and myosin Va through its distinct coiled-coil regions. FEBS Lett..

[29] Matesic L.E., Yip R., Reuss A.E., Swing D.A., O’Sullivan T.N., Fletcher C.F., Copeland N.G., Jenkins N.A. (2001). Mutations in Mlph, encoding a member of the Rab effector family, cause the melanosome transport defects observed in leaden mice. Proc. Natl. Acad. Sci. USA.

[30] Guo D., Jain R., Hwang J.S., Weninger W., Beaumont K.A., Tikoo S. (2020). RAB27A/Melanophilin Blocker Inhibits Melanoma Cell Motility and Invasion. J. Investig. Dermatol..

[31] Wehbe M., Soudja S.M., Mas A. (2012). Epithelial-mesenchymal-transition-like and TGFbeta pathways associated with autochthonous inflammatory melanoma development in mice. PLoS ONE.

[32] Dancea H.C., Shareef M.M., Ahmed M.M. (2009). Role of Radiation-induced TGF-beta Signaling in Cancer Therapy. Mol. Cell. Pharmacol..

[33] Teresa Pinto A., Laranjeiro Pinto M., Patricia Cardoso A. (2016). Ionizing radiation modulates human macrophages towards a pro-inflammatory phenotype preserving their pro-invasive and pro-angiogenic capacities. Sci. Rep..

[34] Li J., Jin Q., Huang F., Tang Z., Huang J. (2017). Effects of Rab27A and Rab27B on Invasion, Proliferation, Apoptosis, and Chemoresistance in Human Pancreatic Cancer Cells. Pancreas.

[35] Liu J., Gong X., Zhu X., Xue D., Liu Y., Wang P. (2017). Rab27A overexpression promotes bladder cancer proliferation and chemoresistance through regulation of NF-kappaB signaling. Oncotarget.

