# Supplementary material for: Overexpression of MLPH in Rectal Cancer Patients Correlates with a Poorer Response to Preoperative Chemoradiotherapy and Reduced Patient Survival
Source: Diagnostics (Basel). 2021 Nov 17;11(11):2132. doi: 10.3390/diagnostics11112132 (PMC8621396; doi:10.3390/diagnostics11112132)
Supplement: Supplementary file 1 [file diagnostics-11-02132-s001.zip › diagnostics-1398606-supplementary.pdf]

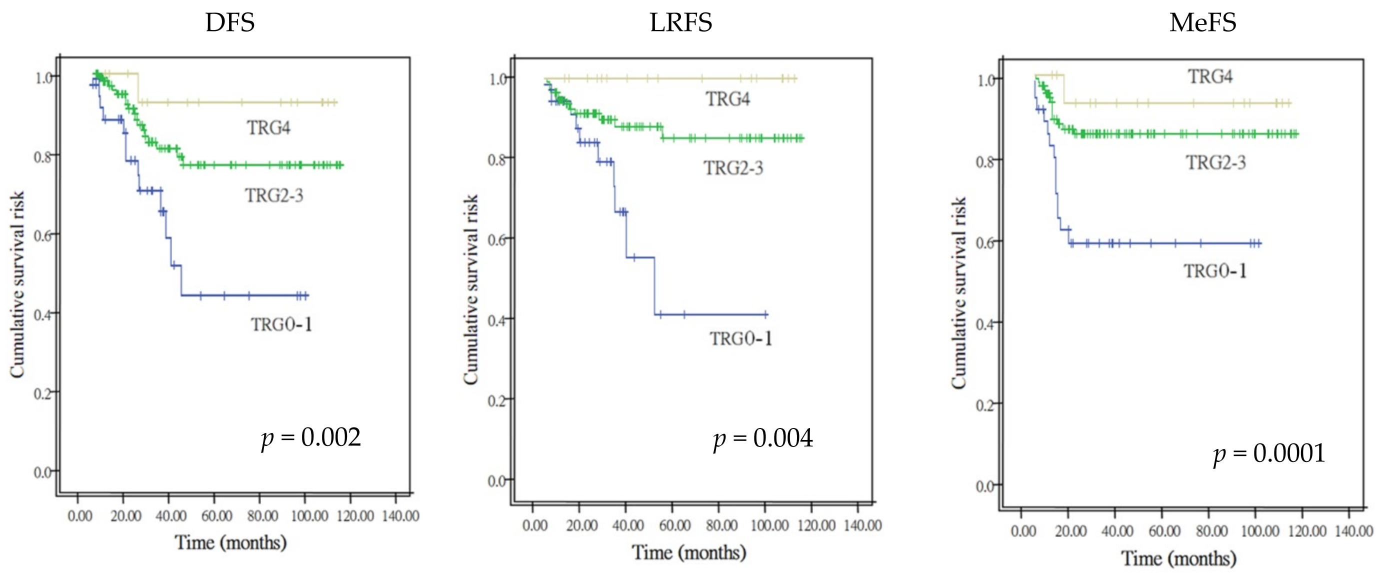

Supplementary Figure S1. Kaplan-Meier curves for 5-year disease free survival (DFS), local recurrence free survival(LRFS) and metastases free survival (MeFS) in relation to tumor regression grading (TRG), (TRG 4 vs. TRG2+3 vs. TRG 0+1).
